# Bidirectional Fermentation of *Monascus* and Ginseng Enhances Pigment and Ginsenoside Rg3 Contents: Process Optimization and Antioxidant Mechanism Analysis

**DOI:** 10.3390/foods15101829

**Published:** 2026-05-21

**Authors:** Luchen Ruan, Xin Zhao, Xin Han, Dongyu Xiang, Yanxiu Xue, Zhuo Chen, Ke Li, Wenrui Du, Zekun Li, Zhi Lu, Xiaole Xia

**Affiliations:** 1 State Key Laboratory of Food Nutrition and Safety, Tianjin University of Science & Technology, Tianjin 300457, China; 2 College of Food Science and Engineering, Tianjin University of Science & Technology, Tianjin 300457, China; 3 Key Laboratory of Industrial Biotechnology, Ministry of Education and School of Biotechnology, Jiangnan University, Wuxi 214122, China; 4 Infinitus (China) Co. Ltd., Guangzhou 510665, China

**Keywords:** functional fermentation, *Monascus ruber*, medicinal and edible plants, water-soluble pigments, antioxidant activity, macrophages, network pharmacology

## Abstract

Oxidative stress is a key contributor to aging and chronic diseases, highlighting the need for safe and effective natural antioxidants. *Monascus* yellow pigments (MYPs) and ginsenoside Rg3 exhibit antioxidant activity, but their applications are restricted by low solubility and limited natural abundance. In this research, a bidirectional liquid fermentation system of *Monascus ruber* using ginseng decoction was established for the simultaneous production of water-soluble MYPs (WSMYPs) and ginsenoside Rg3. Process conditions were optimized to enhance the yields and the antioxidant activity of the system. Antioxidant assays and H_2_O_2_-induced RAW264.7 cell models confirmed that WSMYPs were strongly correlated with antioxidant capacity, with ABTS and DPPH scavenging activities showing 2.28-fold and 3.33-fold increases, respectively, compared to the control. Their combination with Rg3 exerted synergistic protective effects by enhancing the activities of superoxide dismutase (SOD), glutathione peroxidase (GSH-Px), and catalase (CAT). Network pharmacology and molecular docking further revealed that Monapurone C, a representative WSMYP, and Rg3 act through a multi-target, multi-pathway antioxidant network involving signaling pathways such as PI3K-Akt. This study demonstrates a cost-effective strategy for co-producing WSMYPs and Rg3, providing new insights into the value-added utilization of edible and medicinal resources.

## 1. Introduction

Oxidative stress is a major factor that contributes to aging and the development of various chronic diseases [1]. Recently, natural antioxidants from plants and microorganisms have gained attention for their safety and efficacy, becoming a research focus in food science and functional foods [2,3]. *Monascus* species are filamentous fungi widely used in traditional Asian fermented foods, capable of producing *Monascus* pigments (MPs) and other secondary metabolites during fermentation [4]. Among them, *Monascus* yellow pigments (MYPs) are considered ideal natural alternatives to synthetic colorants due to their bright hue and favorable safety profile [5]. Studies have demonstrated that MYPs possess multiple biological activities, including antioxidant, anti-inflammatory, and anti-obesity effects, indicating their application potential in the food and pharmaceutical industries [6,7,8]. Most MYPs are hydrophobic, and current strategies to obtain water-soluble forms often rely on chemical modification or encapsulation, which increases production complexity and cost [9]. During *Monascus* fermentation, MYPs are mainly synthesized through polyketide metabolic pathways and can form water-soluble derivatives under specific conditions. However, the yield of naturally fermented water-soluble MYPs (WSMYPs) remains low, further limiting their practical application [10].

Ginseng, a well-known medicinal food herb, accumulates dozens of ginsenosides [11]. The rare ginsenoside Rg3 exhibits potent antioxidant activity and high bioavailability, but it is nearly absent in raw ginseng and must be generated through thermal processing or microbial transformation [12]. Microbial fermentation has been widely used to convert major ginsenosides into rare forms through enzymatic deglycosylation. Current research on *Monascus*-ginseng fermentation primarily focuses on solid-state methods targeting Rg3 enrichment alone [13]. A key challenge is to develop an efficient and cost-effective fermentation strategy that simultaneously enhances the production of WSMYPs and ginsenoside Rg3. Bidirectional fermentation, a process involving the co-cultivation of medicinal plants and microorganisms to enable both microbial metabolism and transformation of plant constituents, provides a promising strategy [14]. This approach has been successfully applied in *Monascus* and mulberry to increase γ-aminobutyric acid (GABA) and pigment contents [15]. The bidirectional fermentation of *Monascus* and ginseng is not only intended to obtain two groups of bioactive compounds within the same fermentation system but also to explore potential metabolic interactions between the microorganism and plant substrates. Such interactions may stimulate secondary microbial metabolism and facilitate the biotransformation of ginsenosides, thereby improving both compound diversity and production efficiency. Moreover, compared to bidirectional solid-state fermentation, liquid fermentation offers the advantage of direct application without extensive downstream processing.

To further elucidate the antioxidant mechanisms of WSMYPs and ginsenoside Rg3, modern bioinformatics technologies have become increasingly important in the study of functional natural products. Network pharmacology is an emerging AI-assisted approach that integrates biological targets, signaling pathways, and diseases to explore the complex interactions between drugs and their effects [16]. It has become increasingly important for explaining the mechanisms of natural bioactive compounds [17]. In addition, molecular docking is a key computational simulation to predict the binding sites and affinities between small molecules and protein targets, thereby supporting the identification of core target-compound interactions [18]. Several studies have demonstrated that network pharmacology combined with molecular docking is effective in clarifying the mechanisms of bioactive compounds. For instance, researchers have revealed the mechanisms of astaxanthin in improving atherosclerosis and identifying potential targets of berberine in treating hyperlipidemia using this strategy [19]. These findings underscore the reliability and applicability of network pharmacology and molecular simulation in natural product research.

In this study, a bidirectional liquid fermentation system was developed using *Monascus ruber* and ginseng decoction to simultaneously enhance the production of water-soluble Monascus yellow pigments and the rare ginsenoside Rg3, with potential synergistic antioxidant effects. Key parameters, including ginseng addition, inoculum amount, carbon and nitrogen concentrations, were systematically optimized to enhance the co-production of WSMYPs and the rare ginsenoside Rg3. The optimal fermentation conditions were further determined according to antioxidant capacity and protective effects in macrophages. Moreover, network pharmacology and molecular docking were employed to investigate the synergistic antioxidant mechanisms of WSMYPs and Rg3, revealing the multi-target and multi-pathway of their interactions. This work provides a novel approach for high-value utilization of edible and medicinal resources through bidirectional fermentation and offers insights into the underlying mechanisms of their combined antioxidant activity.

## 2. Materials and Methods

### 2.1. Materials and Reagents

*Monascus ruber* strain SFF-4 was obtained from laboratory storage at the State Key Laboratory of Food Nutrition and Safety and the Tianjin University of Science and Technology (Tianjin, China), where the main experiments in this study were conducted. The ginseng was purchased from Beijing Tongrentang (Origin: Changbai Mountain, Beijing, China). The ginsenoside Rg3 standard was obtained from Shanghai Macklin Biochemical Co., Ltd. (Shanghai, China). Medium and chemical analytical-grade reagents were purchased from the Sinopharm Chemical Reagent Co., Ltd. (Shanghai, China). ABTS and DPPH free radical scavenging ability assay kits were purchased from the Beijing Solarbio Science and Technology Co., Ltd. (Beijing, China).

### 2.2. Seed Media and Cultivation

*Monascus ruber* SFF-4 was inoculated onto potato dextrose agar (PDA) slant medium and cultured at 30 °C for 5–7 days, as described in previous studies [20]. The spores were collected with sterile saline to prepare a 1 × 10^7^ spore/mL suspension. The suspension was then inoculated into a liquid seed medium (per liter): glucose 60 g, peptone 25 g, NaNO_3_ 2 g, MgSO_4_·7H_2_O 1 g, K_2_HPO_4_·3H_2_O 2 g, and corn steep liquor 0.5 g (natural pH). Cultivation was carried out at 30 °C and 170 rpm for 2 days on a rotary shaker.

### 2.3. Bidirectional Liquid Fermentation of Monascus and Ginseng

Sliced ginseng (20 g) was soaked and decocted twice after boiling 30 min for the first decoction and 20 min for the second. The combined decoctions and residues were diluted with water to 1 L to obtain the ginseng decoction. To prepare the fermentation medium, 60 g glucose, 25 g peptone, 2 g NaNO_3_, 1 g MgSO_4_·7H_2_O, 2 g K_2_HPO_4_·3H_2_O, and 0.5 g corn steep liquor were added to the decoction. The seed culture was inoculated at 10% (*v*/*v*) into 100 mL of fermentation medium in a 500 mL Erlenmeyer flask and incubated at 30 °C and 170 rpm for 5 days. These were the initial conditions used before optimization.

### 2.4. Preoptimization of Fermentation Process

Four fermentation parameters, including ginseng addition (1–4 wt%), inoculum amount (5–20%), carbon source content (5–20%), and nitrogen source content (1–4%), were investigated for their effects on the yields of the WSMYPs and ginsenoside Rg3. In the single-factor experiments, when one variable was varied, the other variables were kept constant at baseline levels (2 wt% ginseng, 10% inoculum, 10% sucrose, and 2% peptone), which were determined based on preliminary experiments. After fermentation, the broth was centrifuged, and the supernatant was used for pigment analysis.

### 2.5. Experimental Design: Box–Behnken Design for Fermentation Optimization

Based on single-factor experiments, a four-factor, three-level Box–Behnken experimental design was implemented in this study. The ranges of the independent variables were determined based on single-factor experiments as follows: ginseng addition (1–3 wt%), inoculum amount (5–15%), sucrose content (5–15%), and peptone content (1–3%). Each variable was evaluated at three levels in the Box–Behnken design. A total of 27 experimental runs were conducted in triplicate. The response variables were the yields of the WSMYPs and ginsenoside Rg3.

### 2.6. Determination of WSMYPs, Ginsenoside Rg3, and Antioxidant Activity

WSMYP’s concentration (U/mL) was determined spectrophotometrically. After centrifugation, the supernatant was diluted appropriately, and the absorbance was measured at 410 nm. The concentration was calculated as OD_410_ × dilution factor. Ginsenoside Rg3 (mg/mL) was quantified by the HPLC using a C18 column (4.6 × 250 mm, 5 μm; Agilent, Santa Clara, CA, USA) with a PDA detector at 203 nm. The mobile phase consisted of 0.1% phosphoric acid in water (A) and acetonitrile (B), with a linear gradient from 25% to 95% B over 0–25 min at 1.0 mL/min. The column temperature was set at 30 °C and the injection volume was 20 μL [21]. The HPLC chromatogram and standard curve of ginsenoside Rg3 were provided (Figure S1). DPPH and ABTS radical scavenging activities were measured in a 96-well microplate using commercial assay kits from Beijing Solarbio Science and Technology Co., Ltd. (Beijing, China), following the manufacturer’s instructions.

### 2.7. Evaluation of Antioxidant Activity in H_2_O_2_-Induced RAW264.7 Cells

Evaluation of antioxidant activity in H_2_O_2_-induced RAW264.7 cells was performed as previously described, with minor modifications [22]. RAW264.7 macrophages (Manassas, VA, USA) were seeded into the 96-well plates at a density of 1 × 10^4^ cells/well and cultured in complete medium consisting of 89% DMEM, 10% FBS, and 1% penicillin–streptomycin at 37 °C under 5% CO_2_ for 24 h. An H_2_O_2_-induced oxidative damage model was established by exposing cells to different concentrations of H_2_O_2_ for 2 h, and the optimal concentration that reduced cell viability to approximately 50% was determined using the Cell Counting Kit-8 (CCK-8) assay. Cells were then divided into a model group, a positive control group (1 μg/mL vitamin C), and three treatment groups treated with fermentation supernatants obtained from different fermentation. After pretreatment with fermentation products or vitamin C for 0.5 h, the optimal concentration of H_2_O_2_ was added, and cells were incubated for an additional 2 h. Cells were then collected, lysed, and the activities of superoxide dismutase (SOD), glutathione peroxidase (GSH-Px), and catalase (CAT) were measured according to the instructions of the respective assay kits.

### 2.8. Network Pharmacology Analysis and Antioxidant Targets Screening

The targets of key bioactive compounds in the fermentation system were predicted through the SwissTargetPrediction database (http://swisstargetprediction.ch/, accessed on 15 November 2025), while the antioxidant-related targets were retrieved from the GeneCards database (https://www.genecards.org/, accessed on 15 November 2025). Common targets between bioactive compounds and antioxidant activity were identified via Venn analysis. The metabolite-target-function network and protein–protein interaction (PPI) network were constructed using Cytoscape 3.9.1, and core antioxidant targets were screened through topological analysis with the CentiScaPe plugin based on betweenness, degree, and closeness centralities. Finally, Kyoto Encyclopedia of Genes and Genomes (KEGG) pathway enrichment analysis was conducted using the DAVID database (https://david.davidbioinformatics.nih.gov/, accessed on 20 November 2025).

### 2.9. Molecular Docking

The protein structures of the core antioxidant targets were obtained from the Protein Data Bank (https://www.rcsb.org/, accessed on 27 November 2025), and ligand structures were retrieved from PubChem (https://pubchem.ncbi.nlm.nih.gov/, accessed on 27 November 2025). Molecular docking was performed using Schrödinger (Release 2018-4, Schrödinger LLC, New York, NY, USA) [23]. Ligands were prepared using LigPrep by desalting, generating tautomers and ionization states at pH 7.0 using Epik, and minimizing with the OPLS_2005 force field. Protein structures were prepared by assigning bond orders, adding hydrogens (Epik, pH 7.0), removing water molecules, and completing missing side chains and loops using Prime. Energy minimization was performed using the OPLS_2005 force field with a heavy-atom RMSD constraint of 0.30 Å [24]. The binding site was defined as a region within 6 Å of the co-crystallized ligand and confirmed with SiteMap as a single binding site. A receptor grid was generated using the Receptor Grid Generation module. Docking was carried out using the XP (extra precision) mode of Glide with post-docking minimization enabled. Binding poses were analyzed to identify key interactions. The binding energies (kcal/mol) of the ligand–protein complexes were calculated using the Prime MM-GBSA module, based on the OPLS_2005 force field, the VSGB solvent model, and rotamer search algorithms [25]. Glide docking poses were used as input structures for the MM-GBSA calculations, and lower binding energy values indicated stronger binding affinity [26].

### 2.10. Statistical Analysis

Response surface methodology (RSM) was performed using Design-Expert software version 10.0 (Stat-Ease, Inc., Minneapolis, MN, USA) [27], and the significance of the regression model and interaction terms was evaluated by ANOVA. All experiments were performed in triplicate. Statistical significance was performed using IBM SPSS Statistics 24.0 and OriginPro 2021 [28]. Differences among groups were analyzed using ANOVA followed by Duncan’s multiple range test for post hoc comparisons. The value of *p* < 0.05 was considered statistically significant. Pearson correlation analysis was conducted to evaluate the relationships between the concentrations of WSMYPs, ginsenoside Rg3, and antioxidant activities (DPPH and ABTS assays). Protein-ligand interactions and structural characteristics were visualized using PyMOL (version 2.5, Schrödinger LLC, New York, NY, USA).

## 3. Results and Discussion

### 3.1. Main Bioactive Compounds in Monascus-Ginseng Fermentation System and Factors Affecting Production

**Figure 1:**
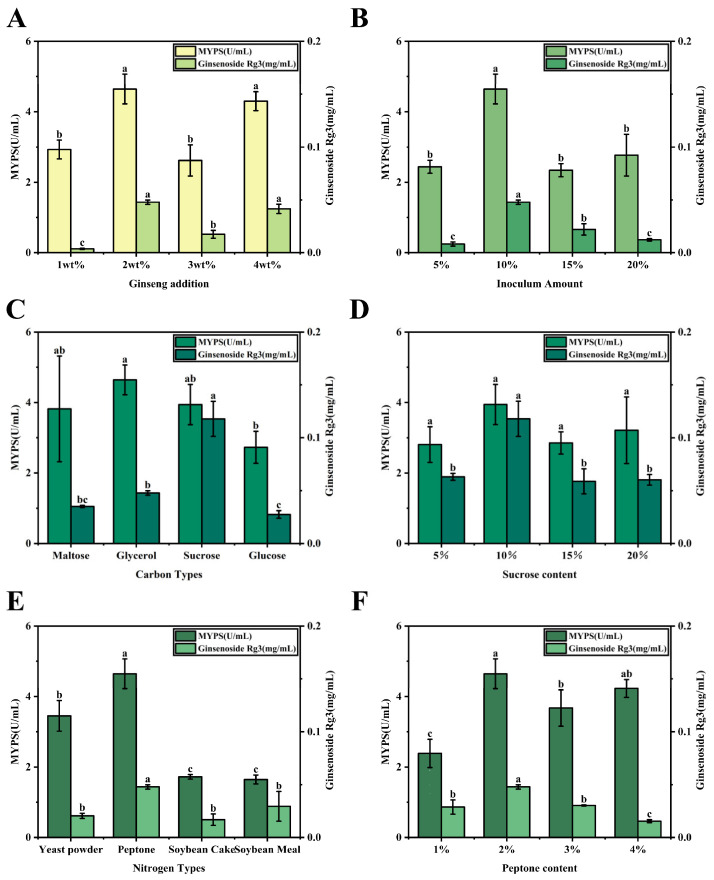
Influence factors on the yields of WSMYPs and ginsenoside Rg3 in the *Monascus*-Ginseng Fermentation System. Influence of ginseng addition on the yields of WSMYPs and ginsenoside Rg3 (**A**). Influence of inoculum amount on the yields of WSMYPs and ginsenoside Rg3 (**B**). Influence of carbon source types and contents on the yields of WSMYPs and ginsenoside Rg3 (**C**,**D**). Influence of nitrogen source types and contents on the yields of WSMYPs and ginsenoside Rg3. Different letters above the bars indicate significant differences at *p* < 0.05 according to Duncan’s multiple range test (**E**,**F**).

WSMYPs and ginsenoside Rg3, the primary antioxidant compounds in the *Monascus*-ginseng fermentation supernatant, were first evaluated in response to key factors. The ginseng addition in the fermentation process had a remarkable influence, with 2 wt% ginseng addition yielding the maximum WSMYPs and ginsenoside Rg3 (Figure 1A). Regarding the inoculum amount, the maximum yields of WSMYPs and ginsenoside Rg3 were observed at 10%. Further increases in inoculum size did not enhance production and instead led to a slight decline in yield (Figure 1B), possibly due to intensified nutrient competition and oxygen limitation caused by excessive biomass [29]. With sucrose identified as the optimal carbon source (Figure 1C), 10% was confirmed to be the most effective concentration (Figure 1D), supplying sufficient energy and carbon while avoiding osmotic inhibition of pigment and ginsenoside synthesis [30]. Moreover, peptone served as the optimal nitrogen source (Figure 1E), with the highest yields observed at a peptone concentration of 2% (Figure 1F). Both insufficient and high nitrogen levels impaired production, likely due to the limited biosynthetic capacity or feedback inhibition [31].

These results indicate the importance of nutrient balance in regulating secondary metabolism during fermentation. Plant-derived substrates contribute metabolic precursors and regulatory compounds that promote pigment biosynthesis. For example, plant extracts have been reported to stimulate MPs production by enhancing polyketide metabolic pathways, while carbon source composition can affect the pigment yield by modulating intracellular redox balance [32,33]. Nitrogen availability also affects secondary metabolite synthesis, as moderate nitrogen limitation is often associated with enhanced pigment biosynthesis. Moreover, ginseng components may facilitate metabolic interactions between plant metabolites and microbial enzymes. Microbial fermentation has been reported to convert major ginsenosides into rare forms through β-glucosidase-mediated deglycosylation [34]. Therefore, the bidirectional fermentation system established in this study likely promotes both pigment biosynthesis and ginsenoside transformation through coordinated metabolic processes. Above all, the optimal conditions obtained from single-factor experiments (2 wt% ginseng, 10% inoculum, 10% sucrose, and 2% peptone) were set as the control group.

### 3.2. Fermentation Process Optimization of Monascus Ruber and Ginseng by RSM

**Figure 2:**
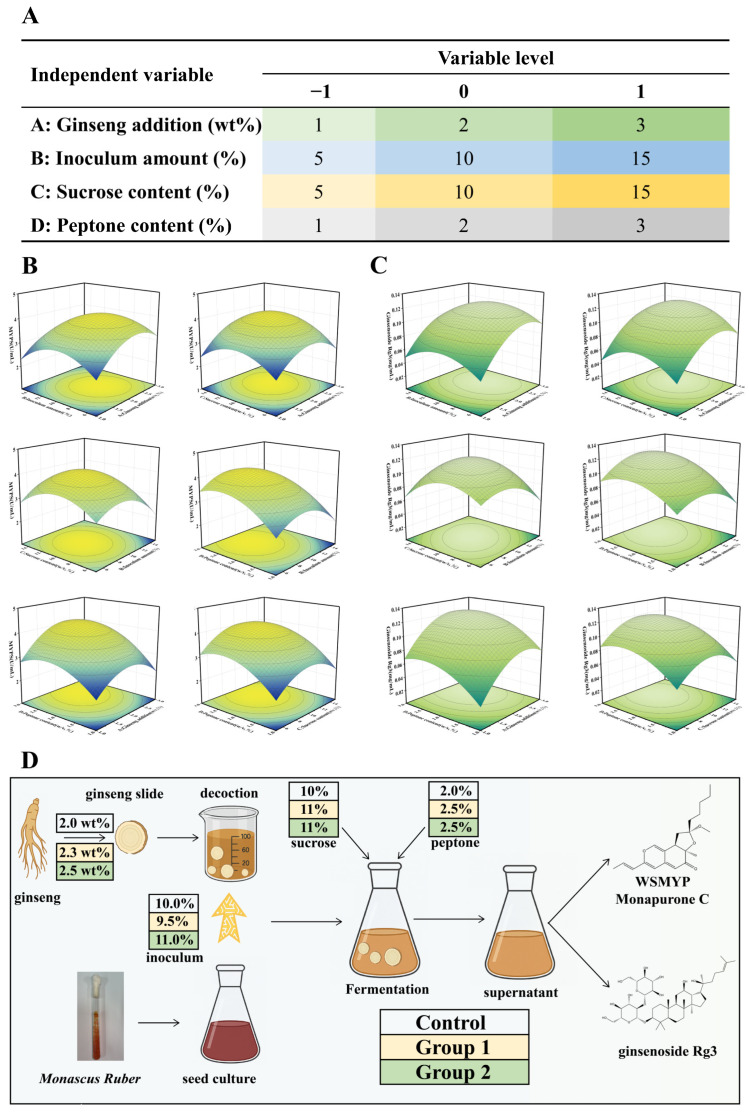
Optimization of WSMYPs and ginsenoside Rg3 Production in the *Monascus*-Ginseng Fermentation Process using RSM. A four-factor, three-level Box–Behnken design (**A**). 3-D response surface plots for yield of WSMYPs (**B**). 3-D response surface plots for yield of ginsenoside Rg3 (**C**). Comparison of different fermentation processes (**D**).

A four-factor, three-level Box–Behnken design was employed in this study (Figure 2A), with variable levels determined based on the above results. The experimental results for WSMYPs production are presented in Table S1, and the corresponding ANOVA results are summarized in Table S2. The model showed the significance and sufficiency of its variables, presented with an F-value of 6.81 and a *p*-value of 0.001. A nonsignificant lack of fit (*p* = 0.9998) indicated a low probability of errors. The adjusted *R*^2^ of 0.7576 and predicted *R*^2^ values of 0.6784 demonstrated that the model accounted for 75.76% of the response variability, supporting its reliability. Some variables did not show significant effects within the tested range, which may be due to the substrate saturation or metabolic regulation. Similarly, the response surface experimental results and ANOVA analysis for ginsenoside Rg3 are presented in Supplementary Tables S3 and S4, respectively, confirming the validity of the model. The relationship between the response variables and the experimental factors was described using a quadratic polynomial equation, where *Y_m_* represents the predicted yield of WSMYPs (U/mL) and *Y_R_* represents the predicted yield of ginsenoside Rg3 (mg/mL) [35]. The independent variables include *A* (ginseng addition, wt%), *B* (inoculum amount, %), *C* (sucrose content, %), and *D* (peptone content, %).


$$Y_{m}=0.6349+0.0571A-0.0289B+0.0304C+0.0987D-0.0191AB+0.0230AC+0.0429AD+0.0297BC+0.0026BD+0.0169CD-0.0646A^{2}-0.0695B^{2}-0.0564C^{2}-0.0392D^{2}\;Y_{R}=0.1181+0.0309A-0.0089B+0.0011C+0.0131D-0.0007AB+0.0135AC-0.0117AD-0.0033BC-0.0017BD-0.0069CD-0.0118A^{2}-0.0418B^{2}-0.0220C^{2}-0.0155D^{2}$$

In addition, 2-D contour plots and 3-D response surface plots were generated to visually illustrate the combined effects of factors on the WSMYPs and ginsenoside Rg3 yields. The interactions of ginseng addition with the inoculum amount, sucrose, and peptone are presented for WSMYPs production and for ginsenoside Rg3 yield (Figure 2B,C and Figure S2). As a result, RSM optimization predicted the maximum yield of WSMYPs as 4.28 U/mL under 2.30 wt% ginseng, 9.48% inoculum, 10.84% sucrose, and 2.52% peptone. The optimal ginsenoside Rg3 yield was 0.13 mg/mL with 2.44 wt% ginseng, 10.83% inoculum, 11.06% sucrose, and 2.55% peptone. For practical purposes, the conditions were set as 2.3 wt% ginseng, 9.5% inoculum, 11% sucrose, and 2.5% peptone for WSMYPs (Group 1), and 2.5 wt% ginseng, 11% inoculum, 11% sucrose, and 2.5% peptone for ginsenoside Rg3 (Group 2). The corresponding fermentation processes are illustrated in Figure 2D. Subsequently, three batches of *Monascus*-ginseng bidirectional liquid fermentation were prepared. The experimental yield of WSMYPs in Group 1 was 4.15 ± 0.06 U/mL, and the experimental yield of ginsenoside Rg3 in Group 2 was 0.14 ± 0.02 mg/mL. The experimental results showed no significant difference from the predicted values, confirming the reliability of the model.

The differences between the optimal conditions for WSMYPs and ginsenoside Rg3 production reflect the distinct metabolic pathway requirements of the pigment biosynthesis and ginsenoside transformation. In particular, ginsenoside Rg3 formation depends on the microbial biotransformation of major ginsenosides in ginseng, which is influenced by enzyme activity and substrate accessibility. Therefore, slight variations in fermentation parameters can redirect metabolic flux toward different products. A coordinated optimization strategy for multiple target products in the fermentation system was adopted by first identifying the optimal conditions for each product separately, followed by evaluation of antioxidant activity to achieve efficient co-production and enhanced functionality of multiple antioxidant compounds.

### 3.3. Antioxidant Properties and Cellular Protective Effects of the Fermentation Process

**Figure 3:**
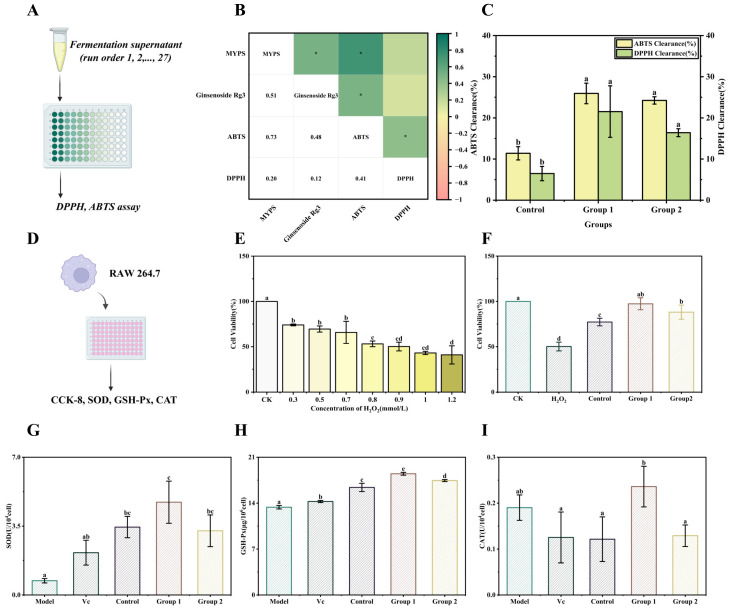
Optimization of the fermentation process based on antioxidant activity and cellular protection. Scheme of the DPPH and ABTS assays for the fermentation supernatants (**A**). Pearson correlation analysis between bioactive compounds and antioxidant activities (**B**). Antioxidant activities of supernatants obtained from different fermentation processes (**C**). Scheme of the protective effect of the fermentation supernatant against oxidative damage in RAW264.7 macrophages (**D**). Optimization of H_2_O_2_ concentration for inducing oxidative damage in RAW264.7 macrophages (**E**). Protective effects of different fermentation groups against H_2_O_2_-induced oxidative damage in RAW264.7 macrophages (**F**). The antioxidant enzyme activities of fermentation groups under H_2_O_2_ exposure. * indicates significant difference at *p* < 0.05 level. Different letters above the bars indicate significant differences at *p* < 0.05 (**G**–**I**).

To determine the optimal process, it was first necessary to identify the main bioactive compounds. The antioxidant activities of fermentation supernatants from 27 experimental runs were determined (Figure 3A). Correlation analysis was then conducted between antioxidant activity and the concentrations of WSMYPs and Ginsenoside Rg3 (Figure 3B). ABTS clearance showed a strong correlation with WSMYPs (*R* = 0.73) and a moderate correlation with Rg3 (*R* = 0.48). A similar pattern was observed for the DPPH scavenging activity, which showed a higher correlation with WSMYPs concentration (*R* = 0.20) than with ginsenoside Rg3 (*R* = 0.12). Overall, WSMYPs contribute more to the antioxidant capacity than ginsenoside Rg3. This conclusion was further confirmed by the antioxidant activities measured under the three fermentation conditions (Figure 3C), with Group 1, which was optimized for the production of WSMYPs exhibiting the highest antioxidant capacity, increasing ABTS and DPPH clearance values by 2.28-fold and 3.33-fold compared to the control, respectively.

To further evaluate the antioxidant potential of the fermentation product, an H_2_O_2_-induced oxidative damage model in RAW264.7 cells was established (Figure 3D). When treated with 0.9 mmol/L H_2_O_2_, cell viability decreased to 50.19 ± 4.82%, which was selected as the optimal modeling condition (Figure 3E). Treatment with different concentrations of the fermentation broth showed no cytotoxicity (cell viability > 80%), indicating suitability for subsequent experiments (Figure S3). On this basis, CCK-8 assays revealed that H_2_O_2_ significantly reduced cell viability, whereas fermentation broth treatment markedly improved cell survival. Consistent with the in vitro antioxidant assays, Group 1 showed a clear protective effect, with cell morphology and density similar to the blank control (Figure 3F and Figure S3).

Analysis of antioxidant enzyme activities showed that the treatments with the fermentation broths significantly enhanced the SOD, GSH-Px, and CAT activities in H_2_O_2_-treated cells. The highest levels were observed in Group 1, with SOD at 4.72 ± 1.07 U/10^4^ cells, GSH-Px at 18.48 ± 0.22 U/10^6^ cells, and CAT at 0.23 ± 0.04 U/10^4^ cells (Figure 3G–I). These findings demonstrate that the fermentation product effectively alleviates oxidative damage, and Group 1 showed higher antioxidant activity than Group 2, mainly due to the synergistic contribution of the WSMYPs. Previous studies have shown that MYPs possess conjugated structures that enable efficient scavenging of free radicals and inhibition of oxidative reactions. Meanwhile, although the direct contribution of ginsenoside Rg3 to radical scavenging appears moderate, its biological effects in cells may enhance endogenous antioxidant defenses. The observed improved activities of SOD, GSH-Px, and CAT indicate that the fermentation products not only neutralize reactive oxygen species directly but also activate cellular antioxidant systems. To further clarify their underlying antioxidant mechanisms, network pharmacology analysis was applied.

### 3.4. Network Pharmacology-Based Antioxidant Targets Screening

**Figure 4:**
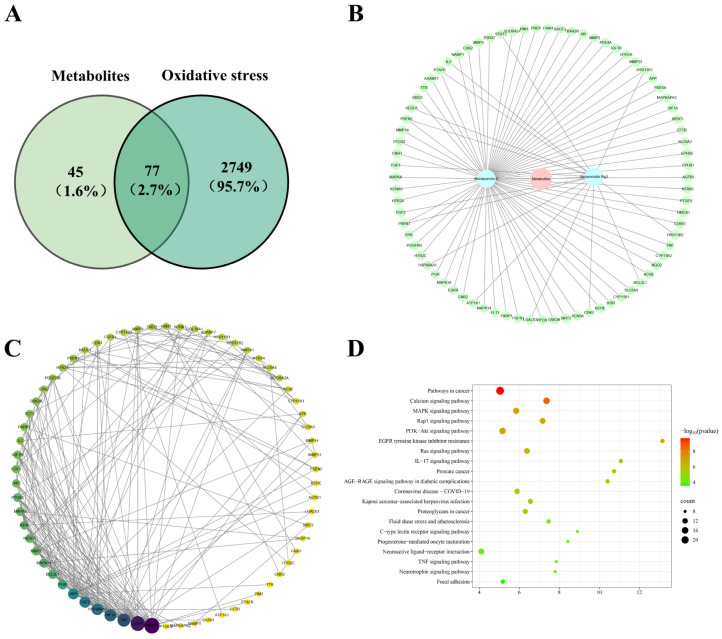
Network pharmacology analysis of Monapurone C and ginsenoside Rg3 in antioxidant pathways. Venn diagram of compound targets in Monapurone C and ginsenoside Rg3 and antioxidant-related targets (**A**). Network diagram of metabolite-target-activity (**B**). Protein–protein interaction network of intersection antioxidant targets (**C**). The top 20 significantly enriched pathways by KEGG enrichment analysis (*p* < 0.05) (**D**).

Network pharmacology integrates systems biology with network analysis to explore drug-target interactions and predict potential mechanisms of action [36]. In this work, network pharmacological analysis was used to reveal the potential antioxidant mechanisms of WSMYPs and ginsenoside Rg3 (InChIKey: RWXIFXNRCLMQCD-JBVRGBGGSA-N). Since WSMYPs are complex mixtures, Monapurone C (InChIKey: HDEPPKKHVHQOFH-QHJSOZPLSA-N), a well-characterized and representative compound, was selected for further analysis [37]. A total of 122 potential targets were identified via the SwissTargetPrediction database (probability > 0). In addition, 2826 antioxidant-related targets were retrieved from the GeneCards database (score > 5.0), yielding 77 overlapping targets (Figure 4A). To visualize the interactions, a metabolite-target-activity network was constructed, consisting of 80 nodes (1 sample node, 2 metabolite nodes, 77 antioxidant targets) and 83 edges (Figure 4B). This network revealed multiple interactions between the metabolites and targets, highlighting that the antioxidant effect arises from the synergistic action of Monapurone C and ginsenoside Rg3. These findings align with the synergistic characteristics of multiple components and targets described in previous network pharmacology analysis [38]. Indeed, the biological effects of dietary bioactive compounds are mainly the result of such synergistic interactions [39]. A representative example is the significant synergistic antioxidant effect observed between curcumin and (−)-epicatechin [40].

To identify the core antioxidant targets, a protein–protein interaction (PPI) network was constructed using 77 antioxidant-related targets of active compounds via the STRING database (confidence score > 0.7) and visualized with Cytoscape 3.9.1 (Figure 4C). Topological analysis was performed using the CentiScape plugin. The resulting PPI network contained 66 nodes and 200 edges, indicating that Monapurone C and ginsenoside Rg3 in fermentation can exert antioxidant effects through multiple targets and pathways. The average values of betweenness, degree, and closeness centrality of nodes were 0.03, 6.06, and 0.34, respectively. A total of 11 antioxidant targets exceeded the average values of more than three topological parameters (Table 1). Based on the size of the circle and the depth of the color, five highly correlated core antioxidant targets were obtained: signal transducer and activator of transcription 3 (STAT3), epidermal growth factor receptor (EGFR), tumor necrosis factor (TNF), hypoxia-inducible factor 1 subunit alpha (HIF1A), and heat shock protein 90 alpha family class A member 1 (HSP90AA1). Among them, EGFR, TNF, and HIF1A were associated with the antioxidant activity of Monapurone C, whereas STAT3 and HSP90AA1 were related to ginsenoside Rg3 (Figure S4). Antioxidant defense relies on the enzymatic and non-enzymatic systems to maintain redox homeostasis [41]. The five core targets contribute to this defense through coordinated regulation. As key transcription factors, STAT3 and HIF1A reduce the accumulation of reactive oxygen species (ROS) by directly inducing antioxidant enzymes, including SOD and CAT [42,43]. EGFR and TNF regulate ROS production and defense via PI3K/Akt-Nrf2 and NF-κB pathways, respectively [44,45]. HSP90AA1 stabilizes critical signaling proteins, thereby maintaining the structural and functional integrity of the cellular antioxidant network [46]. Furthermore, KEGG enrichment analysis of the 77 targets of Monapurone C and ginsenoside Rg3 revealed involvement in 100 pathways, notably including calcium, MAPK, Rap1, and PI3K-Akt signaling pathways. The PI3K-Akt pathway receives upstream signals from EGFR and HSP90AA1, activating Nrf2-mediated transcription of antioxidant genes [47]. These findings outline a multilayered defense network, revealing the biological basis by which Monapurone C and ginsenoside Rg3 cooperatively eliminate reactive oxygen species through cross-level coordination of targets and pathways.

### 3.5. Molecular Docking of the Bioactive Compounds and Core Antioxidant Targets

**Figure 5:**
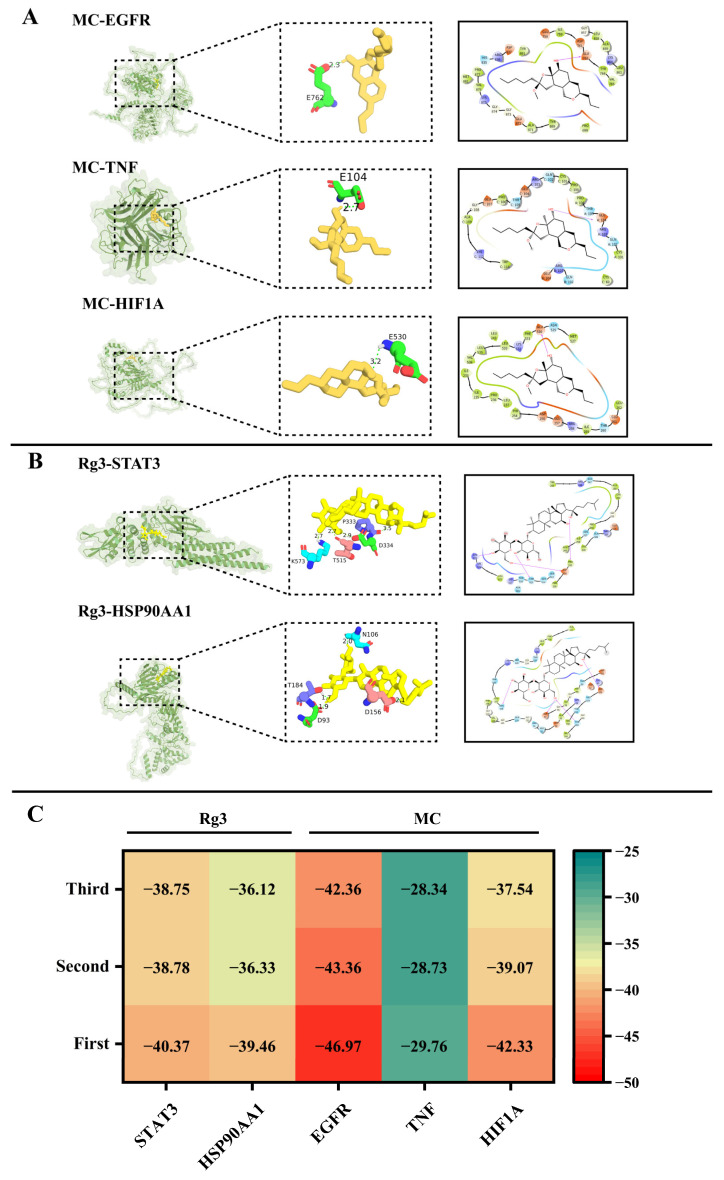
Molecular docking analysis of compounds and core antioxidant targets. Binding conformations of Monapurone C with EGFR, TNF, and HIF1A (**A**). Binding conformations of ginsenoside Rg3 with STAT3 and HSP90AA1 (**B**). Binding energy between core antioxidant targets and the two compounds across different trials (**C**).

Molecular docking is widely used to simulate protein–ligand interactions for drug development and mechanism prediction of natural compounds based on the induced-fit theory. To further investigate the potential mechanisms of Monapurone C and ginsenoside Rg3, docking was performed by Schrödinger to evaluate their binding modes and affinities with antioxidant targets. Monapurone C interacted with EGFR via a single hydrogen bond at the E667 residue (2.3 Å), with TNF at the E104 residue (2.7 Å), and with HIF1A at the E530 residue (3.2 Å) (Figure 5A). Rg3 formed four hydrogen bonds with both STAT3 and HSP90AA1 (Figure 5B). The docking scores further supported strong binding energies, with values of −46.97, −29.76, and −42.33 kcal/mol for Monapurone C with EGFR, TNF, and HIF1A, and −40.37 and −39.46 kcal/mol for Rg3 with STAT3 and HSP90AA1, respectively (Figure 5C). All docking scores were below −29 kcal/mol, indicating robust binding activity between the ligands and receptors. These interactions suggest that both compounds bind stably within the target protein pockets, contributing to their synergistic antioxidant effects [48]. Through hydrogen-bond networks, they achieve targeted regulation of antioxidant pathways and cross-pathway coordination, providing a structural basis for the integrated antioxidant mechanism of the *Monascus*–ginseng fermentation.

These docking results are consistent with the network pharmacology predictions and provide structural evidence for the multi-target antioxidant mechanism of the fermentation products. Similar integrative approaches combining network pharmacology and molecular docking have been widely applied to elucidate the mechanisms of natural bioactive compounds. The strong binding affinities observed between Monapurone C, ginsenoside Rg3, and the identified antioxidant targets further suggest that these compounds may regulate oxidative stress through multiple signaling pathways. Such multi-component and multi-target characteristics are typical for natural products and fermented functional foods, highlighting the potential health benefits of the *Monascus*-ginseng fermentation system developed in this study.

## 4. Conclusions

In conclusion, we established a bidirectional liquid fermentation process to enhance the bioactive compounds in *ginseng* and *Monascus*. This process significantly improved the antioxidant activity of the fermentation products and demonstrated good potential for industrial application. Correlation analysis and in vitro assays indicated that WSMYPs were the primary contributors to antioxidant activity, while ginsenoside Rg3 exerted a synergistic effect, together forming the antioxidant mechanism. Furthermore, network pharmacology and molecular docking revealed that Monapurone C and Rg3 act synergistically through multiple components, targets, and pathways. The main antioxidant targets, including STAT3, EGFR, TNF, HIF1A, and HSP90AA1, regulate ROS balance and redox homeostasis via PI3K-Akt, MAPK, calcium, and Rap1 signaling pathways. All findings in this study offer the material basis and mechanism underlying the synergistic enhancement of antioxidant activity by *Monascus* and ginseng and provide new insights for developing highly antioxidant functional foods.

## Figures and Tables

**Table 1:** The topological characteristics of the main antioxidant targets.

No.	Target	Betweenness	Degree	Closeness Centrality
1	STAT3	0.195971	25	0.507813
2	EGFR	0.148985	23	0.488722
3	TNF	0.133719	19	0.471014
4	HIF1A	0.043273	17	0.439189
5	HSP90AA1	0.132874	16	0.454545
6	FGF2	0.083637	15	0.439189
7	APP	0.216658	14	0.448276
8	MAPK14	0.045923	10	0.389222
9	PTGS2	0.034005	9	0.401235
10	NR3C1	0.110497	9	0.414013
11	PARP1	0.062433	7	0.363128

## Data Availability

The original contributions presented in this study are included in the article and Supplementary Material. Further inquiries can be directed to the corresponding authors.

## Supplementary Materials

The following supporting information can be downloaded at: https://www.mdpi.com/article/10.3390/foods15101829/s1, Figure S1: High performance liquid chromatography analysis of ginsenoside Rg3 (A). Standard curve of ginsenoside Rg3 (B); Figure S2: 2-D contour plots for yield of WSMYPs (A). 2-D contour plots for yield of ginsenoside Rg3 (B); Figure S3: Cytotoxic effects of different groups on RAW264.7 macrophages (A). Cell morphology of RAW264.7 macrophages treated with different groups (B); Figure S4: Network pharmacology analysis of Monapurone C in antioxidant pathways (A). Network pharmacology analysis of ginsenoside Rg3 in antioxidant pathways (B); Table S1: Response surface experiment results of WSMYPs; Table S2: ANOVA for the quadratic model of WSMYPs through optimization of the fermentation process; Table S3: Response surface experiment results of ginsenoside Rg3; Table S4: ANOVA for the quadratic model of ginsenoside Rg3 through optimization of the fermentation process.
