# Supplementary material for: Bidirectional Fermentation of Monascus and Ginseng Enhances Pigment and Ginsenoside Rg3 Contents: Process Optimization and Antioxidant Mechanism Analysis
Source: Foods. 2026 May 21;15(10):1829. doi: 10.3390/foods15101829 (PMC13205446; doi:10.3390/foods15101829)
Supplement: Supplementary file 1 [file foods-15-01829-s001.zip › foods-4196798-supplementary.pdf]

<u>**Supporting Material**</u>

This file includes:

Figure S1 to S4

Tables S1 to S4

Supplementary figures captions:

Figure S1 High performance liquid chromatography analysis of ginsenoside Rg3 (A). Standard curve of ginsenoside Rg3 (B).

Figure S2 2-D contour plots for yield of WSMYPs (A). 2-D contour plots for yield of ginsenoside Rg3 (B).

Figure S3 Cytotoxic effects of different groups on RAW264.7 macrophages (A). Cell morphology of RAW264.7 macrophages treated with different groups (B).

Figure S4 Network pharmacology analysis of Monapurone C in antioxidant pathways (A). Network pharmacology analysis of ginsenoside Rg3 in antioxidant pathways (B).

Supplementary tables captions:

Table S1 Response surface experiment results of WSMYPs.

Table S2 ANOVA for the quadratic model of WSMYPs through optimization of the fermentation process.

Table S3 Response surface experiment results of ginsenoside Rg3

Table S4 ANOVA for the quadratic model of ginsenoside Rg3 through optimization of the fermentation process.

Supplementary Figures

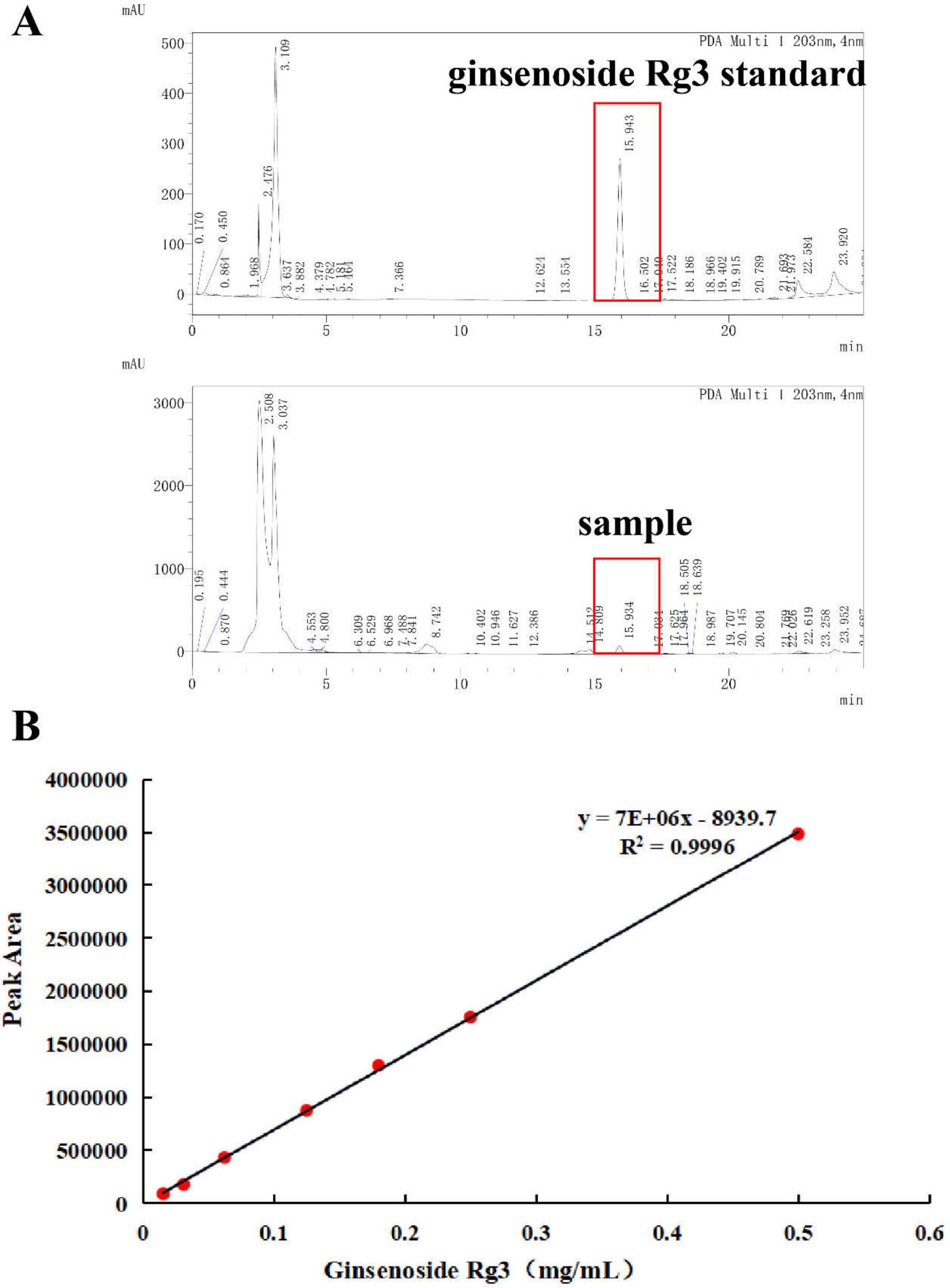

Figure S1

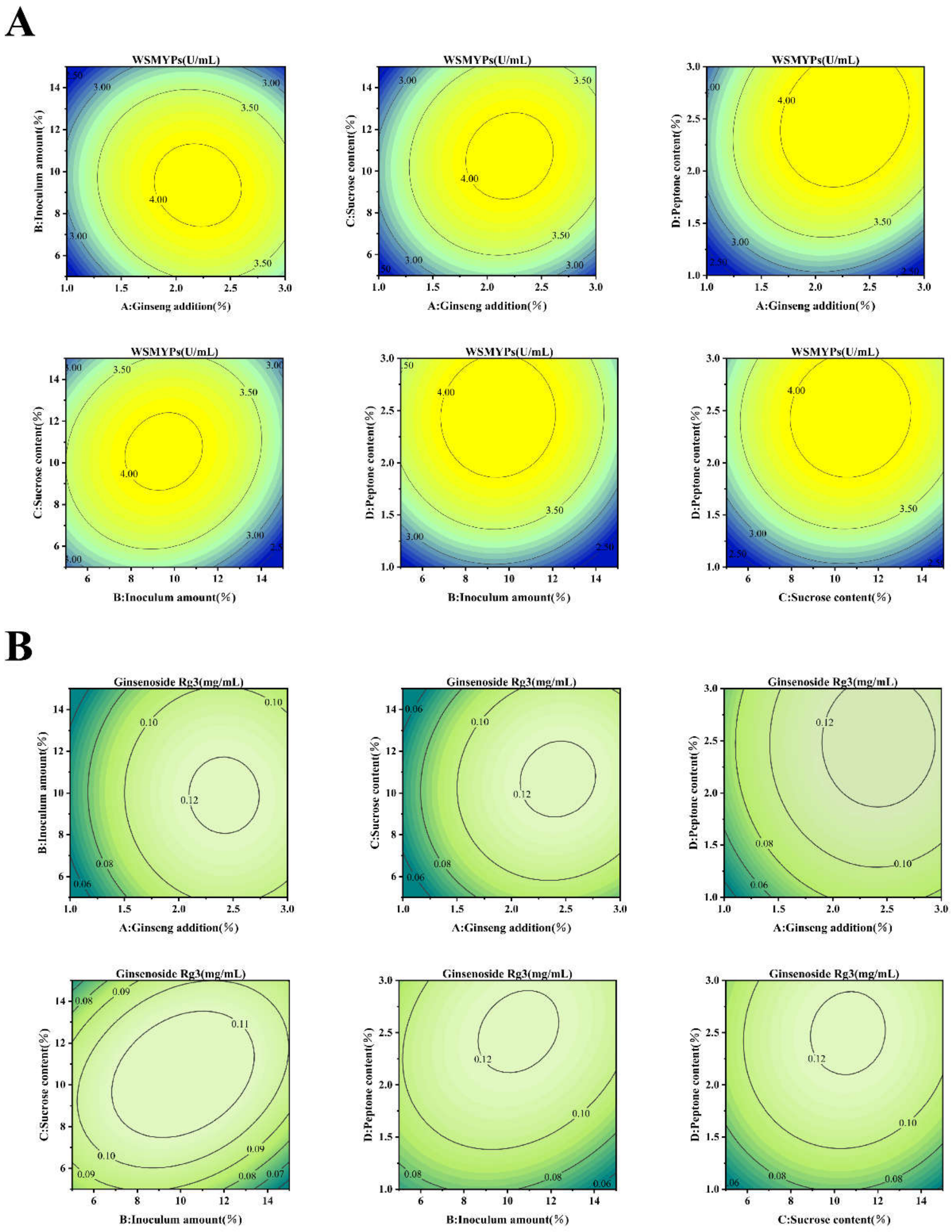
A
WSMYPs(U/mL)
A:Ginseng addition(%)
B:Inoculum amount(%)
C:Sucrose content(%)
D:Peptone content(%)
B
Ginsenoside Rg3(mg/mL)

Figure S2

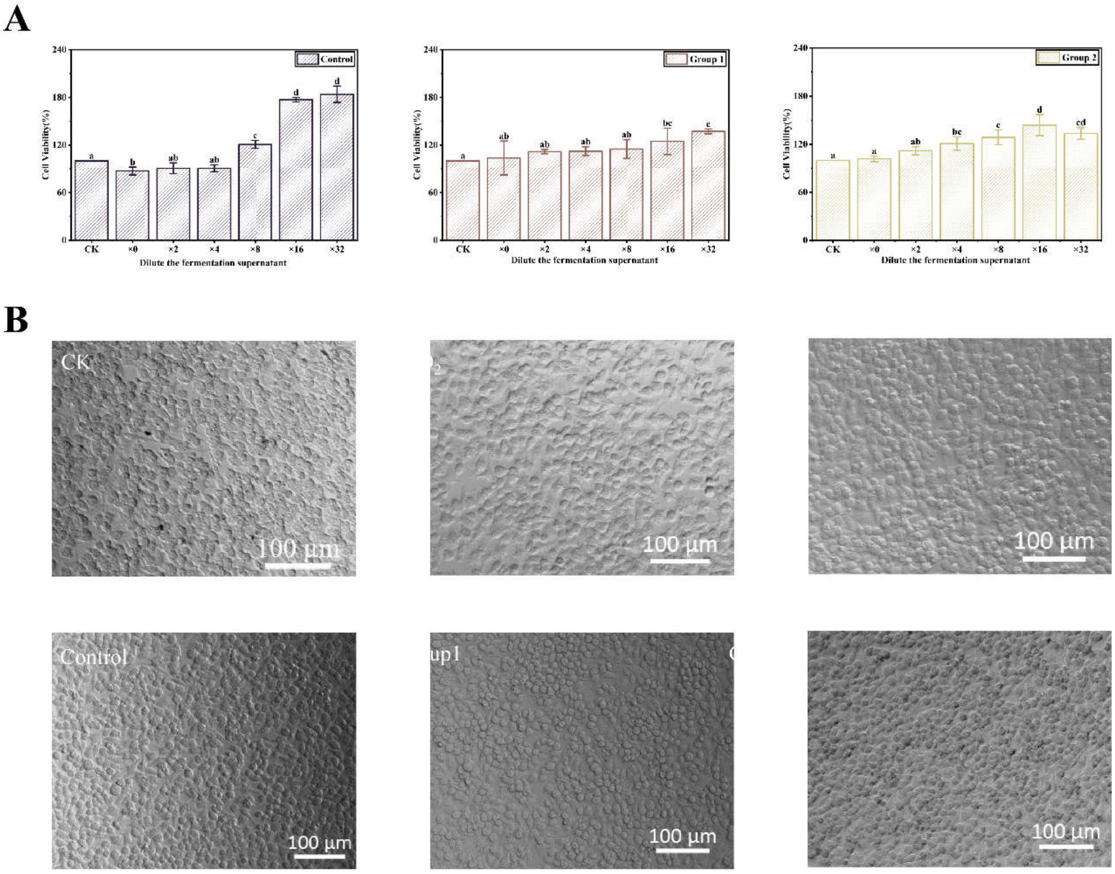
A
Control
Group 1
Group 2
Cell Viability(%)
Dilute the fermentation supernatant
CK
×0
×2
×4
×8
×16
×32
B
CK
Control
100 µm

Figure S3

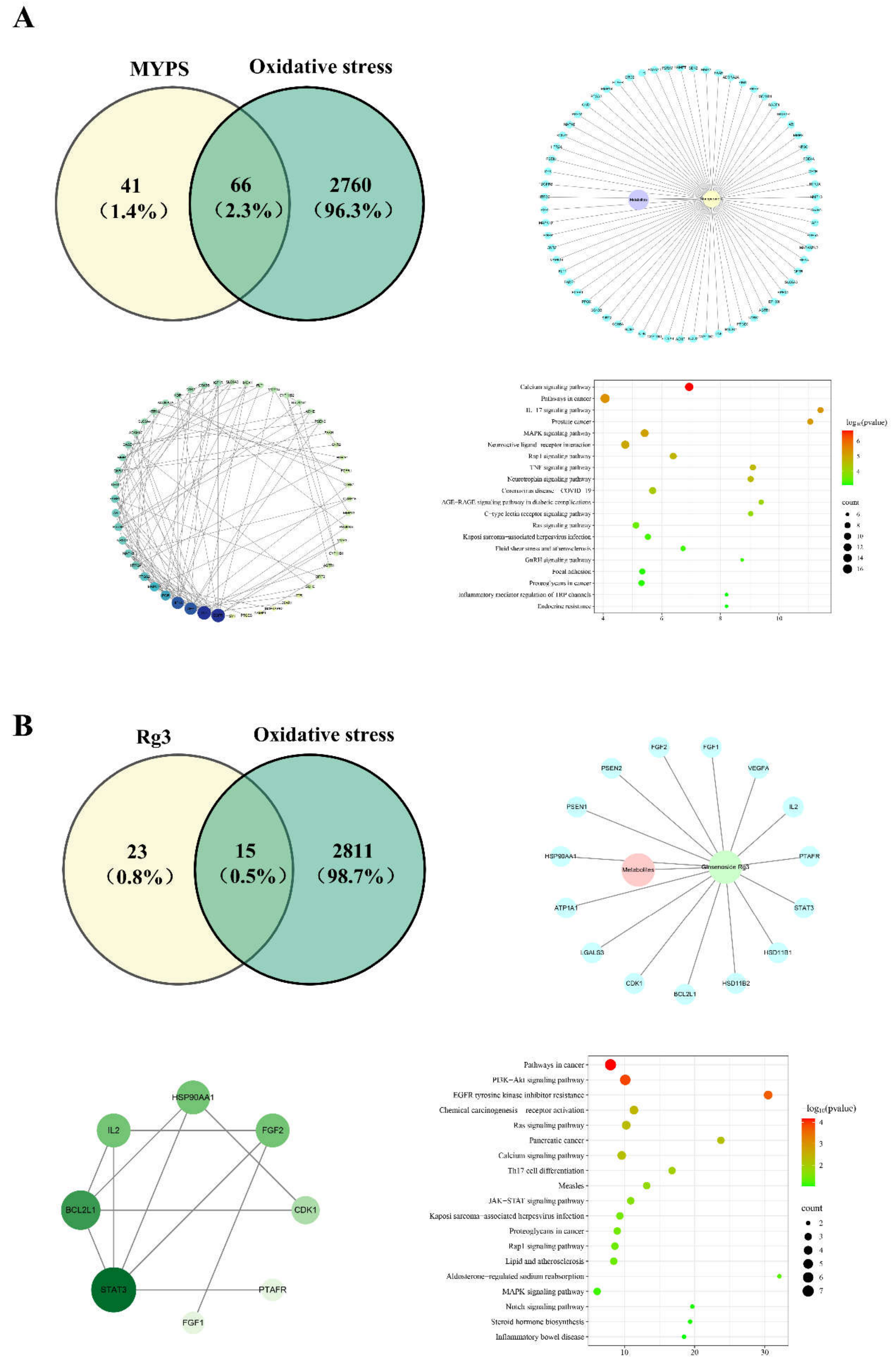
A
MYPS
Oxidative stress
41
（1.4%）
66
（2.3%）
2760
（96.3%）
Calcium signaling pathway
Pathways in cancer
IL−17 signaling pathway
Prostate cancer
MAPK signaling pathway
Neuroactive ligand−receptor interaction
Rap1 signaling pathway
TNF signaling pathway
Neurotrophin signaling pathway
Coronavirus disease − COVID−19
AGE−RAGE signaling pathway in diabetic complications
C−type lectin receptor signaling pathway
Ras signaling pathway
Kaposi sarcoma−associated herpesvirus infection
Fluid shear stress and atherosclerosis
GnRH signaling pathway
Focal adhesion
Proteoglycans in cancer
Inflammatory mediator regulation of TRP channels
Endocrine resistance
log10(pvalue)
count
B
Rg3
Oxidative stress
23
（0.8%）
15
（0.5%）
2811
（98.7%）
FGF2
FGF1
PSEN2
VEGFA
PSEN1
IL2
HSP90AA1
Metabolites
Ginsenoside Rg3
PTAFR
ATP1A1
STAT3
LGALS3
HSD11B1
CDK1
BCL2L1
HSD11B2
BCL2L1
CDK1
PTAFR
FGF1
Pathways in cancer
PI3K−Akt signaling pathway
EGFR tyrosine kinase inhibitor resistance
Chemical carcinogenesis − receptor activation
Ras signaling pathway
Pancreatic cancer
Calcium signaling pathway
Th17 cell differentiation
Measles
JAK−STAT signaling pathway
Kaposi sarcoma−associated herpesvirus infection
Proteoglycans in cancer
Rap1 signaling pathway
Lipid and atherosclerosis
Aldosterone−regulated sodium reabsorption
MAPK signaling pathway
Notch signaling pathway
Steroid hormone biosynthesis
Inflammatory bowel disease
log10(pvalue)
count

Figure S4

Supplementary Tables

Table S1 Response surface experiment results of WSMYPs

	**A**	**B**	**C**	**D**	**Predicted value**	**Experimental value**
1	1	5	10	2	2.37	2.43
2	3	5	10	2	3.17	3.11
3	1	15	10	2	2.28	2.24
4	3	15	10	2	2.59	2.44
5	2	10	5	1	2.15	2.16
6	2	10	15	1	2.35	2.25
7	2	10	5	3	3.09	3.09
8	2	10	15	3	3.56	3.46
9	1	10	10	1	2.16	2.09
10	3	10	10	1	2.25	2.14
11	1	10	10	3	2.78	2.87
12	3	10	10	3	3.80	3.85
13	2	5	5	2	2.83	2.63
14	2	15	5	2	2.12	2.11
15	2	5	15	2	2.79	2.78
16	2	15	15	2	2.83	3.01
17	1	10	5	2	2.37	2.38
18	3	10	5	2	2.61	2.78
19	1	10	15	2	2.39	2.32
20	3	10	15	2	3.26	3.35
21	2	5	10	1	2.40	2.58
22	2	15	10	1	1.99	2.08
23	2	5	10	3	3.41	3.43
24	2	15	10	3	3.14	3.08
25	2	10	10	2	4.08	3.23
26	2	10	10	2	4.08	4.66
27	2	10	10	2	4.08	4.34

Table S2 ANOVA for the quadratic model of WSMYPs through optimization of the fermentation process

**Source**	**Sum of Squares**	**df**	**Mean Square**	**F-value**	**p-value**	
Model	10.85	14	0.7750	6.81	0.0010	significant
A-Ginseng addition	0.9278	1	0.9278	8.15	0.0145	
B-Inoculum amount	0.3367	1	0.3367	2.96	0.1112	
C-Sucrose content	0.3389	1	0.3389	2.98	0.1101	
D-Peptone content	3.50	1	3.50	30.76	0.0001	
AB	0.0584	1	0.0584	0.5129	0.4876	
AC	0.1003	1	0.1003	0.8806	0.3665	
AD	0.2147	1	0.2147	1.89	0.1949	
BC	0.1394	1	0.1394	1.22	0.2903	
BD	0.0049	1	0.0049	0.0430	0.8391	
CD	0.0191	1	0.0191	0.1681	0.6891	
A2	2.86	1	2.86	25.10	0.0003	
B2	2.95	1	2.95	25.91	0.0003	
C2	2.55	1	2.55	22.43	0.0005	
D2	1.91	1	1.91	16.79	0.0015	
**Residual**	1.37	12	0.1139			
Lack of Fit	0.2435	10	0.0244	0.0434	0.9998	not significant
Pure Error	1.12	2	0.5615			
**Cor Total**	12.22	26				
**S.D**	0.3375		**R^2**	0.8881		
**Mean**	2.85		**Adj. R^2**	0.7576		
**Coeffcient of variance**	11.85		**Pred. R^2**	0.6784		

Table S3 Response surface experiment results of ginsenoside Rg3

	A	B	C	D	Predicted value	Experimental value
1	1	5	10	2	0.05	0.05
2	3	5	10	2	0.09	0.10
3	1	15	10	2	0.05	0.04
4	3	15	10	2	0.09	0.09
5	2	10	5	1	0.05	0.05
6	2	10	15	1	0.06	0.07
7	2	10	5	3	0.08	0.08
8	2	10	15	3	0.10	0.11
9	1	10	10	1	0.03	0.04
10	3	10	10	1	0.08	0.08
11	1	10	10	3	0.07	0.06
12	3	10	10	3	0.11	0.11
13	2	5	5	2	0.08	0.08
14	2	15	5	2	0.05	0.06
15	2	5	15	2	0.06	0.06
16	2	15	15	2	0.09	0.09
17	1	10	5	2	0.04	0.05
18	3	10	5	2	0.08	0.08
19	1	10	15	2	0.04	0.04
20	3	10	15	2	0.10	0.08
21	2	5	10	1	0.07	0.06
22	2	15	10	1	0.05	0.04
23	2	5	10	3	0.09	0.09
24	2	15	10	3	0.11	0.11
25	2	10	10	2	0.12	0.13
26	2	10	10	2	0.12	0.11
27	2	10	10	2	0.12	0.11

Table S4 ANOVA for the quadratic model of ginsenoside Rg3 through optimization of the fermentation process

**Source**	**Sum of Squares**	**df**	**Mean Square**	**F-value**	**p-value**	
Model	0.0176	14	0.0013	12.52	< 0.0001	significant
A-Ginseng addition	0.0062	1	0.0062	61.52	< 0.0001	
B-Inoculum amount	2.377E-06	1	2.377E-06	0.0236	0.8804	
C-Sucrose content	0.0003	1	0.0003	2.68	0.1275	
D-Peptone content	0.0040	1	0.0040	40.03	<0.0001	
AB	4.703E-06	1	4.703E-06	0.0468	0.8324	
AC	0.0001	1	0.0001	0.7960	0.3898	
AD	2.267E-06	1	2.267E-06	0.0225	0.8831	
BC	0.0007	1	0.0007	6.79	0.0230	
BD	0.0003	1	0.0003	3.40	0.0902	
CD	0.0000	1	0.0000	0.3946	0.5416	
A2	0.0040	1	0.0040	39.47	< 0.0001	
B2	0.0024	1	0.0024	23.62	0.0004	
C2	0.0033	1	0.0033	32.79	< 0.0001	
D2	0.0019	1	0.0019	18.87	0.0010	
**Residual**	0.0012	12	0.0001			
Lack of Fit	0.0010	10	0.0001	0.8256	0.6620	not significant
Pure Error	0.0002	2	0.0001			
**Cor Total**	0.0188	26				
**S.D**	0.0100		**R2**	0.9359		
**Mean**	0.0772		**Adj. R2**	0.8612		
**Coeffcient of variance**	12.99		**Pred. R2**	0.6749		
